# Ethnobotanical Knowledge Is Vastly Under-Documented in Northwestern South America

**DOI:** 10.1371/journal.pone.0085794

**Published:** 2014-01-09

**Authors:** Rodrigo Cámara-Leret, Narel Paniagua-Zambrana, Henrik Balslev, Manuel J. Macía

**Affiliations:** 1 Departamento de Biología, Universidad Autónoma de Madrid, Madrid, Spain; 2 Herbario Nacional de Bolivia, Universidad Mayor de San Andrés, La Paz, Bolivia; 3 Department of Bioscience, Aarhus University, Aarhus, Denmark; New York State Museum, United States of America

## Abstract

A main objective of ethnobotany is to document traditional knowledge about plants before it disappears. However, little is known about the coverage of past ethnobotanical studies and thus about how well the existing literature covers the overall traditional knowledge of different human groups. To bridge this gap, we investigated ethnobotanical data-collecting efforts across four countries (Colombia, Ecuador, Peru, Bolivia), three ecoregions (Amazon, Andes, Chocó), and several human groups (including Amerindians, mestizos, and Afro-Americans). We used palms (Arecaceae) as our model group because of their usefulness and pervasiveness in the ethnobotanical literature. We carried out a large number of field interviews (n = 2201) to determine the coverage and quality of palm ethnobotanical data in the existing ethnobotanical literature (n = 255) published over the past 60 years. In our fieldwork in 68 communities, we collected 87,886 use reports and documented 2262 different palm uses and 140 useful palm species. We demonstrate that traditional knowledge on palm uses is vastly under-documented across ecoregions, countries, and human groups. We suggest that the use of standardized data-collecting protocols in wide-ranging ethnobotanical fieldwork is a promising approach for filling critical information gaps. Our work contributes to the Aichi Biodiversity Targets and emphasizes the need for signatory nations to the Convention on Biological Diversity to respond to these information gaps. Given our findings, we hope to stimulate the formulation of clear plans to systematically document ethnobotanical knowledge in northwestern South America and elsewhere before it vanishes.

## Introduction

In 1992, the Convention on Biological Diversity (CBD) established that signatory nations are obliged to (i) respect, preserve, and maintain traditional knowledge relevant to conservation and sustainable use of biological diversity, (ii) promote wide application of traditional knowledge, and (iii) encourage equitable sharing of benefits arising from the use of traditional knowledge [1]. Changes in lifestyle brought by globalization have led to an abandonment of traditional practices with a concurrent loss of related knowledge [2], [3], and in countries rich in biological and cultural diversity, to the extinction of indigenous groups with small populations [4]. Therefore, it is necessary that the signatory nations of CDB react to these threats and, in line with the Aichi Biodiversity Targets [5], evaluate how much traditional knowledge exists across their territories, identify the ethnic groups whose knowledge, belief and practices have been studied in that respect, quantify how much of the extant traditional knowledge of their (indigenous) inhabitants has been registered in the literature, and determine which methods are the most efficient for salvaging remaining knowledge.

Ethnobotany documents traditional knowledge about plants and can be used to engage policy-makers and development planners in designing appropriate strategies for the conservation of cultures and cultural knowledge related to their useful plants [6]. Nonetheless, most ethnobotanical publications to date have been limited to one or few indigenous groups or use categories (e.g., medicinal plants or edible plants) and have mostly been carried out at local scales; as a consequence, comprehensive cross-scale knowledge is lacking. Moreover, little is known about the coverage of past efforts and thus about how well the ethnobotanical literature documents the overall traditional knowledge of each ethnic group. Evaluating the efficiency of past efforts to document ethnobotanical knowledge can bridge these gaps and shed light on which methods could be the most time-effective for collecting the remaining information.

Palms (Arecaceae) are an excellent model group for evaluating past ethnobotanical efforts in South America because they are among the most commonly mentioned plant families in the ethnobotanical literature [7]–[12], they constitute keystone resources in the subsistence of local people [13]–[15], and they are taxonomically well understood [16]–[21]. Recently, an exhaustive literature review of palm uses in northwestern South America (Colombia, Ecuador, Peru, Bolivia) assembled available data from works published between 1947 and 2009 [15]. For the first time, palm use patterns were analyzed at different scales, including: ecoregions (the Amazon basin, the Andes, and the Chocó), countries (Colombia, Ecuador, Peru, and Bolivia), and human groups (Amerindians, mestizos, and Afro-Americans).

**Figure 1:**
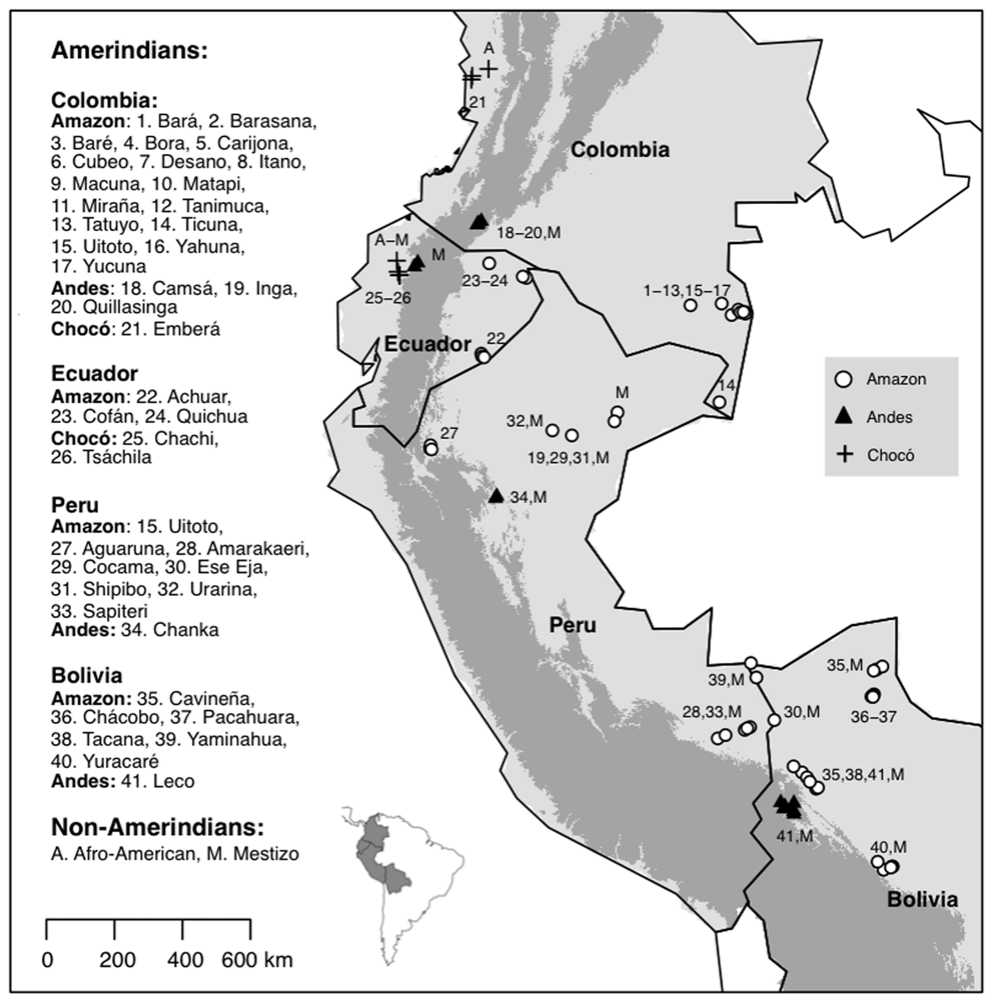
Communities and ecoregions in northwestern South America where palm ethnobotanical data were gathered.

In this study, we explored how effectively ethnobotanists have documented traditional knowledge about palm uses across northwestern South America. We did so by comparing the coverage and quality of palm ethnobotanical data reported in the literature and reviewed in Macía et al. [15] against field data from a very large dataset obtained through systematic interviews collected during 18 months of fieldwork in the same study area (Fig. 1). This work is, to our knowledge, the first study in which ethnobotanical information from the literature is validated through intensive fieldwork in the same study region. Specifically, we ask the following five questions:

1. *How well were ethnobotanical uses of palms documented in the literature when compared with data from intensive fieldwork in northwestern South America?* Because the existing literature covers a longer time span and more localities, we predicted that it would contain more information than we could collect in our fieldwork, especially for the Amazon ecoregion because of the great number of published works based on studies in that region [15]. For the Andes and Chocó, we expected equal coverage of data derived from the literature and from fieldwork because these ecoregions have been less studied in the past in terms of palm ethnobotany [15].
2. *How does the documented knowledge of Amerindians compare to that of other human groups (mestizos and Afro-Americans) at large scales?* We expected fewer information gaps in the data relating to Amerindian groups because they are more studied than the others [15]. Because a common assumption is that Amerindians possess a larger body of knowledge than other groups [22]–[24], we expected to find large differences in total knowledge between Amerindians and the mestizo and Afro-American populations.
3. *How do data from the literature compare with data from fieldwork for the 22 Amerindian groups for which both sources are available?* We expected that our fieldwork would include more information than is presented in the literature because our interviews with individuals from each Amerindian group were systematically based on a standard protocol, and we had large sample sizes and an exclusive focus on only one plant family.
4. *How well were different use categories documented in the literature, and does the existing ethnobotanical literature match data from our large dataset from fieldwork in the ranking of most important use categories?* Because of the rural nature of the communities visited during fieldwork and their isolation from markets, we hypothesized that the most important use categories would be the same in the existing literature and in the data obtained from recent fieldwork and that these categories would be *Construction*, *Cultural*, *Human food*, and *Utensils and tools* [15].
5. *Are the species described as the most useful in the literature also the ones that emerge as the most useful in the data derived from our field study?* We expected to find similarity in the ranking of the most useful palms between both datasets, with a small set of species being clearly more useful than others.

## Results

### Geographic Distribution

**Figure 2:**
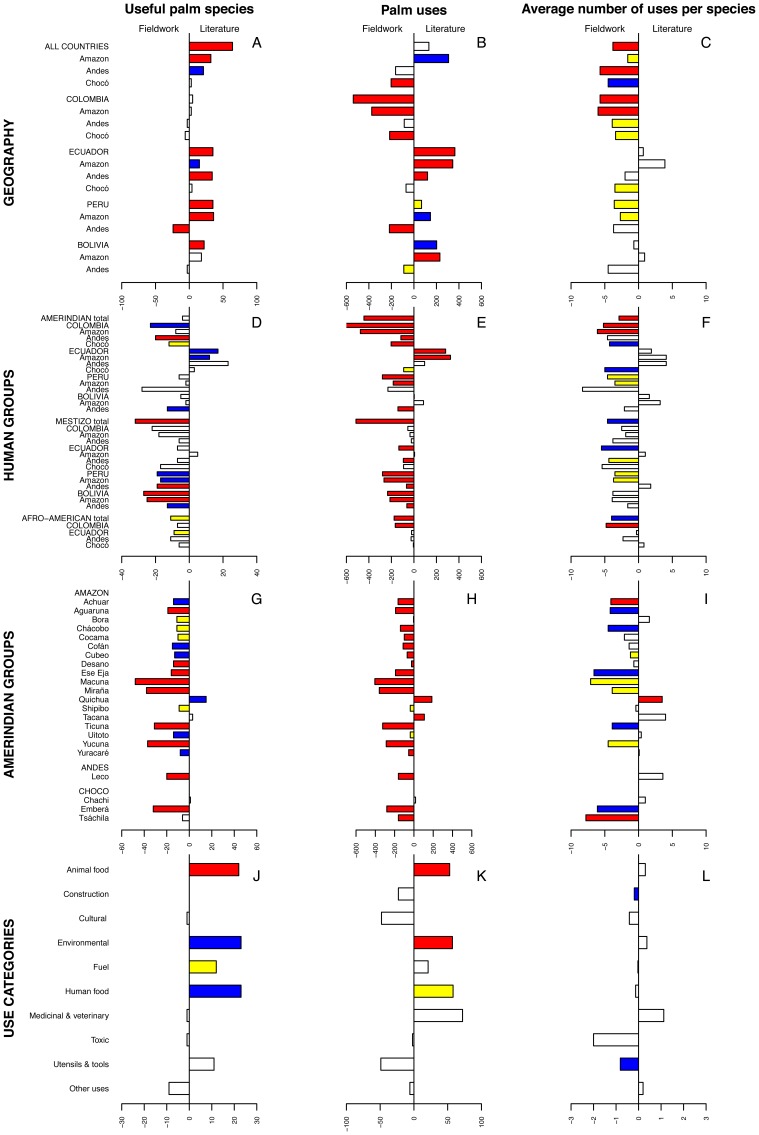
Difference in ethnobotanical data-collecting efforts between fieldwork and literature for three indicators at multiple scales, including Geography (*A–C*), Human groups (*D–F*), Amerindian groups (*G–I*), and Use categories (*J–L*). For each of the three indicators (useful palm species, palm uses, average number of uses per palm species), a bar under “Fieldwork” indicates that the fieldwork yielded more data whereas a bar under “Literature” indicates that the literature reported more data. Significance values are expressed by bar color: red, *P*<0.001; blue, *P*<0.01; yellow, *P*<0.05; and white: not significant.

In the 2201 interviews completed in the western Amazon, the Andes, and the Chocó, a total of 87,886 use reports were gathered and could be classified into 2262 different palm uses and 140 useful palm species (Table 1). Overall, we found that the Amazon was the best-documented ecoregion in the literature because it was the only place where the literature reported both more useful species and more palm uses than the fieldwork (Fig. 2*A–C*). However, in all three ecoregions, fieldwork identified higher average numbers of uses per palm species. Greater information gaps were found in the literature from the Andes and Chocó, where fieldwork in addition to identification of a higher average number of uses per palm species also yielded more palm uses than the literature.

**Table 1:** Ethnobotanical data collected by fieldwork in northwestern South America.

Country	Ecoregion	No. Useful species	No. Palmuses	Average number ±SDof uses per species	No. Palm use reports	No. Informants	No. Communities	No. Amerindian groups interviewed(% total Amerindian groups)*
**All**	Total	**140**	**2262**	**16.1±16.9**	**87,886**	**2201**	**68**	**41 (37)**
	Amazon	102	1664	16.3±16.2	62,749	1277	48	33 (33)
	Andes	47	507	10.8±9.0	13,488	610	13	5 (71)
	Chocó	49	550	11.2±9.1	11,649	314	7	3 (50)
**Colombia**	Total	**100**	**1353**	**13.5±12.8**	**25,879**	**548**	**15**	**21 (47)**
	Amazon	67	990	14.8±12.7	16,070	205	9	17 (44)
	Andes	21	121	5.8±6.5	1959	169	3	3 (60)
	Chocó	44	411	9.3±7.3	7850	174	3	1 (25)
**Ecuador**	Total	**68**	**572**	**8.4±7.3**	**14,654**	**460**	**12**	**5 (42)**
	Amazon	47	331	7.0±5.5	8622	147	6	3 (38)
	Andes	18	119	6.6±4.7	2233	173	2	–
	Chocó	26	237	9.1±7.1	3799	140	4	2 (50)
**Peru**	Total	**61**	**717**	**11.8±10.6**	**28,329**	**613**	**15**	**9 (19)**
	Amazon	57	625	11.0±9.3	24,236	523	13	8 (17)
	Andes	28	237	8.5±6.1	4093	90	2	1 (100)
**Bolivia**	Total	**40**	**453**	**11.3±10.1**	**19,024**	**580**	**26**	**7 (39)**
	Amazon	36	372	10.3±8.4	13,821	402	20	7 (41)
	Andes	16	167	10.4±8.3	5203	178	6	1 (50)

Total number of Amerindian groups was obtained from Lewis et al. [51].

Ecuador was the best-documented country in existing studies, with more information reported in these publications than resulted from fieldwork across most of its ecoregions (Fig. 2*A–C*). Colombia, in contrast, had the greatest information gaps in the literature, and fieldwork in that country gathered more information on palm uses, higher average number of uses per palm species, and almost as many useful species as had been documented in published reports. Information gaps in Peru and Bolivia were moderate, with the literature reporting more information than fieldwork for the Amazon but less for the Andean ecoregion.

### Human Groups

Altogether, in the three human groups analyzed, fieldwork overall generated more ethnobotanical information than the literature offered (Fig. 2*D–F*). Regarding Amerindian groups, the only relatively well-documented country in the literature was Ecuador. In contrast, the remaining countries and especially Colombia had great information gaps. Mestizos were even more under-documented than Amerindian groups, and except for the Ecuadorian Amazon, fieldwork with mestizos gathered more information for all indicators than the literature offered. Among Afro-Americans, we also found considerable information gaps, and our fieldwork documented more than the published reports did for most indicators in most regions.

We collected information from fewer Amerindian groups than those represented in the literature in the Amazon and the Chocó ecoregions but more in the Andes. Only in Colombia did field interviews document as many Amerindian groups as the literature data. Fieldwork gathered information from 19 Amerindian groups for which there was no ethnobotanical palm information in the literature (Colombia: Bará, Barasana, Baré, Camsá, Carijona, Inga, Itano, Matapí, Quillasinga, Tanimuca, Tatuyo, Yahuna; Peru: Amarakaeri, Chanka, Urarina, Sapiteri; Bolivia: Cavineña, Pacahuara, Yaminahua). Nevertheless, the Amerindians were the best-studied human group in both fieldwork and literature, followed by mestizos and Afro-Americans.

### Amerindian Groups Represented in Fieldwork and Literature

Our fieldwork yielded information for 41 Amerindian groups, 22 of which were represented in the literature (Fig. 2*G–I*). In 19 of the 22 Amerindian groups common to both datasets, fieldwork discovered significantly more ethnobotanical knowledge than what was available in published reports. In the remaining three groups (Chachi, Quichua, Tacana), the opposite was true. Most cases were statistically significant (Fig. 2*G–I*).

### Use Categories

We found that literature documented more useful palm species and palm uses and higher average numbers of palm uses per species than fieldwork in about half of all use categories (Fig. 2*J–L*). In addition, the findings of both data sources agreed in showing the same use categories as the most diverse in terms of numbers of useful species and palm uses. Thus, the categories *Construction*, *Cultural*, *Human food*, and *Utensils and tools* had the highest number of useful palm species in both datasets and also the highest number of palm uses along with *Medicinal and veterinary*. Similarly, *Cultural*, *Medicinal and veterinary*, and *Utensils and tools* had the highest average numbers of uses per palm species in both datasets. Differences in the average number of uses per palm species between literature and fieldwork were minimal, and only significantly higher in fieldwork for *Construction* and *Utensils and tools*.

### The Most Useful Palm Species

In general terms, the species with the highest relative importance in our fieldwork data matched those in the literature findings (Table S1). Twelve (80%) of the top fifteen species were shared between the two datasets (fieldwork and literature) in the Amazon ecoregion, ten (67%) in the Chocó, and six (40%) in the Andes. Overall, the fieldwork documented more palm uses per species in the Andes (14 species) and Chocó (11), but the literature reported more in the Amazon (12). In the Amazon, *Oenocarpus bataua* had the highest number of palm uses in both sources. In the Andes, *Attalea phalerata* was the species with the highest number of palm uses in fieldwork, but in the literature, it was *Bactris gasipaes* var. *gasipaes*. In the Chocó, *Iriartea deltoidea* was the palm with the greatest number of uses according to findings from the field, but in the literature, it was *Cocos nucifera*. None of the most useful species are considered threatened in IUCN Red Lists. Our analysis of fieldwork- and literature-derived data revealed the presence of a group of palms (*Bactris gasipaes* var. *gasipaes*, *Oenocarpus bataua*, and *Iriartea deltoidea*) that combined high relative importance with a wide geographic range encompassing all three ecoregions.

## Discussion

Palm use knowledge is clearly understudied for all human groups across ecoregions and countries in our study area in northwestern South America (Colombia, Ecuador, Peru, Bolivia). Our hypothesis that a literature review would reveal more data for all indicators was only partially confirmed for the Amazon ecoregion. Although this review reported more useful species, fieldwork documented a higher average number of uses per palm species. A long-standing history of Amazonian ethnobotanical research with records from 200+ publications [15] likely accounts for the high level of documentation of palm uses in the Amazon. The factors resulting in the high number of uses reported for each species in the fieldwork data are most likely the standardized data-collecting protocol where each interviewee is consistently asked about the uses of the different palm parts of all species reported by expert informants, the large sample sizes, and the stratified sampling of different age and gender groups [25], [26]. Some major advantages of this protocol are that the data collected is quantitative, completely comparable and suitable for statistical analyses. One drawback of using our standardized protocol is that regional approach is cost demanding, so it may not be a feasible option for all researchers. However, one step to overcome this drawback is to increase collaboration and data sharing practices among researchers.

In contrast to the Amazon, in the Andes and Chocó, we found large information gaps. These ecoregions have received little attention from ethnobotanists in comparison to the Amazon, and except for Ecuador, the number of ethnobotanical works relating to them is low. Coupled with the fact that for over half of Andean and Chocó palm species no uses have been recorded [15], our findings underscore the need to increase research in these ecoregions.

Of all countries, we found Colombia had the greatest information gaps in the literature. These gaps were not surprising, given that in the literature review Colombia was the country with the second lowest number of references and also the country with second lowest proportion of indigenous groups with documented palm uses [15]. Furthermore, Colombia ranks among the countries in Latin America with the lowest number of peer-reviewed publications on ethnobiology [27]. Notwithstanding, we can think of at least four ways that it is possible to bridge these gaps in the coming decades, namely by (i) creating more research groups that specialize in ethnobotany, (ii) stimulating students to publish in peer-reviewed journals, (iii) increasing the frequency of events and/or associations that buttress the development of the field, and (iv) promoting international collaborations.

In agreement with our hypothesis, we found that palm ethnobotanical knowledge among Amerindian groups exceeded that of mestizos and Afro-Americans. Although this result is congruent with previous reports in northwestern South America [15], [22], [23], differences may appear larger than they are because of our sample’s bias towards Amerindian informants. When comparing similar sample sizes among mestizos and Amerindians in the Peruvian and Bolivian Amazon, for example, the numbers of useful species and palm uses registered for each group were similar, confirming that mestizos also have profound ethnobotanical knowledge [24], [28]–[30]. Similarly in the Chocó, knowledge among Afro-Americans was close to that of Amerindians. A prolonged history of contact with Amerindians–favoring information exchange [31] and associated with the process of trial and error leading to innovative knowledge–can explain high levels of knowledge among Afro-Americans. Clearly, more research with mestizo and Afro-American groups is needed, not only because their knowledge may be comparable to that of Amerindians but also because they have been largely neglected in the palm ethnobotanical literature [15], [32]. In addition, mestizos and Afro-Americans have large and widespread populations that would permit regional comparisons. In the case of Chocó Afro-Americans, a study of their palm uses would be interesting not least because they reside in an area that harbors the richest palm flora in South America [33].

Our research demonstrated that in ethnobotanical terms, Amerindian groups have yet to be studied in-depth. Not only are almost 50% of the Amerindian groups in northwestern South America unrepresented in studies [15], but also the data that exist for many groups are fragmentary. The remarkably low values for palm uses in the literature dataset across all indicators in comparison to our fieldwork data may be attributed to a combination of several factors, including (i) paucity of monographs that study all useful species of one ethnic group [11], [34], (ii) a greater emphasis on palms that provide cash income [35]–[41], and (iii) the lack of a systematic methodology for gathering information.

The consensus found between the literature and the fieldwork data in ranking *Human food*, *Construction*, *Cultural*, and *Utensils and tools* as the most important use categories confirms that across northwestern South America, most uses revolve around subsistence practices [15]. Furthermore, the agreement between both data sources in pinpointing the same species as most useful suggests that across space and time, local people have been consistent in the valuation of a set of palm species as keystone resources. Of the identified most useful species in northwestern South America, most are canopy palms with large fruits, in line with previous findings suggesting that usefulness is positively correlated with salient characteristics such as stem height [42], [43] and/or fruit diameter [44].

Because our conclusions rely on data about palms, which rank among the best-researched plant families in ethnobotany, we should expect traditional knowledge about all plant families across ecoregions, countries, and human groups to be even more under-documented. Nevertheless, our assessment indicates that regional-scale research and application of a standard method can efficiently confront these shortcomings. Replicating large-scale assessments will be vital for implementing the CBD and for achieving the 20 Aichi Biodiversity Targets stated at the 10th Conference of the Parties to the CBD [5]. Necessary steps include commitments of signatories to respecting traditional knowledge and integrating it into the implementation of the Convention (target 18) and to improving, sharing, and applying by 2020 the knowledge about, among others, biodiversity and its values (target 19). The first of these targets can be met only through national actions that protect, preserve, and promote the traditional knowledge of indigenous and local communities. The second calls for an increased amount of and improvement in the quality of information concerning the values of biodiversity, many of which are poorly recognized or understood. Our work is a contribution to fulfilling both targets and represents a first step towards a clear plan for systematically documenting traditional knowledge in northwestern South America and elsewhere before it vanishes.

## Methods

### Study Area and Human Groups

The research was conducted in the western Amazon basin; the tropical Andes biodiversity hotspot of Colombia, Ecuador, Peru, and Bolivia; and the Chocó biodiversity hotspot of Colombia and Ecuador (Fig. 1). This area may be the richest part of the world for angiosperms [45] and ranks second in palm diversity [46]. Overall, nearly 195 palm species have been reported for the Amazon, 135 for the Andes and 105 for the Chocó [47]. We defined the three ecoregions in our study area as (i) the Amazon: lands east of the Andes below 1000 m; (ii) the Andes: montane forests above 1000 m; and (iii) the Chocó: humid forests along the Pacific littoral of Colombia and northwestern Ecuador. The three human groups in our sample were (i) the original Amerindian population; (ii) mestizos, who are people of mixed origin whose parents belong to different races and who generally are white–indigenous; and (iii) Afro-Americans, who are Black Americans of African ancestry. Definitions of the different ecoregions and human groups follow Macía et al. [15].

### Data Collection

We collected information about palm uses from two sources: (i) interviews (n = 2201) made during 18 months (May 2010 to December 2011) of fieldwork and (ii) the published ethnobotanical literature (n = 255) [15]. In the text, for simplicity, we call these two sources of data “fieldwork” and “literature.” The criteria in Macía et al. (2011) for selecting papers form the literature were (pp.: 465–466): “International and national publications for each of the four countries, including ethnographical publications with data on the uses of palms, when species identification was clear. Three categories of publications were selected. The first included publications based on original data gathered from fieldwork, including scientific papers, books, monographs, book chapters, and graduate, masters and doctoral theses. The second category included review publications for which we checked that data had not been previously published, in order to avoid duplication of information. The third type included publications based on herbarium material which included ethnobotanical information that was not included in any publications.” There were 202 works from the Amazon, 40 from the Andes and 38 from the Chocó [15]. It is possible that some works in the literature were not incorporated into our revision. However, we maintain that the bibliographic revision was very exhaustive because it was conducted by palm ethnobotanists who have worked extensively in Ecuador, Peru and Bolivia (H. Balslev, M. Macía & N. Paniagua) and are thus very familiar with the literature of these countries. Furthermore, we consulted the palm specialists from Colombia (Gloria Galeano) and from Perú (Betty Millán & Joaquina Albán) and they supplemented our revision with data from less accessible references published in their respective countries.

Before starting fieldwork, we developed a standard protocol to gather ethnobotanical data [25], [26]. In each of the three ecoregions, we visited communities belonging to at least two ethnic groups. Communities were selected on the basis of having (i) a uniform ethnic composition within the community, (ii) different accessibility to markets between the communities, and (iii) access to (mature) forests for harvesting palm resources. Ethnobotanical data were collected with two types of participants: expert informants, of whom we interviewed 1–7 in each community (in total n = 171); and general informants, of whom we interviewed 1–85 in each community (in total n = 2030). Selection of experts was through consensus during a community meeting. In communities too large for gathering all villagers, such as in Andean sites with populations exceeding 1000 inhabitants, experts were recruited by asking several general informants to recommend their most knowledgeable peers. Experts were mostly men (78%) and older than 40 years (70%). Walks in the field with each of them were performed to document palm uses and to compile a list of the vernacular names of as many palm species as possible. Once experts were interviewed, we used the list of compiled vernacular names as the basis for interviews with general informants. We selected general informants in each community (or group of communities belonging to one ethnic group when there were fewer than 87 informants in one community) in a stratified manner to have a representative sample of gender (women, n = 1107; men, n = 1094) and age classes (18–30 years, 28%; 31–40 years, 23%; 41–50 years, 20%; 51–60 years, 13%; >60 years, 16%). Interviews were conducted in Spanish or when needed with a local interpreter. Palm species were identified in the field, and specimens were collected when our field identification needed confirmation. Voucher specimens (n = 203) are deposited in the herbaria of AAU, AMAZ, COL, LPB, QCA, and USM, acronyms according to Thiers [48]. We followed the World Checklist of Palms to unify nomenclature [49].

### Data Analyses

Data were analyzed at the species level with the exception of *Bactris gasipaes*, where we differentiated the cultivated var. *gasipaes* from the wild var. *chichagui.* We classified each palm use report into one of ten use categories and subcategories following the Economic Botany Data Collection Standard [49], with some modifications proposed by Macía et al. [15]. A subcategory is a more detailed classification of each use category. For instance, the Human food category is divided into four subcategories: Beverages, Food, Food additives and Oils. For a list of subcategories see Macía et al. (pp. 467–469) [15]. Modifications consisted in changes in the grouping- and naming- of categories (termed Level 1 states in Cook [50]). These changes included the grouping of Food additives into Human food, the inclusion of Vertebrate poisons and Non-vertebrate poisons into a new category called Toxic uses, and the exclusion of Bee plants and Gene sources. Name changes included the use of the term Other uses instead of Invertebrate foods, and of Cultural uses instead of Social uses. To compare data from the literature with data from fieldwork, we used three indicators of ethnobotanical data-collecting effort: (i) number of useful palm species; (ii) number of palm uses, defined as the use of a palm part from a given species associated with a use category and a use subcategory; and (iii) average number of uses per palm species. We contrasted these indicators across: (i) ecoregions, (ii) countries, (iii) human groups, (iv) Amerindian groups present in both datasets, (v) use categories, and (vi) the most useful palm species. The relative importance index was calculated for each species to highlight the most useful palm species in each ecoregion following Macía et al. [15]: RI = NUC+NT, where NUC is the number of use categories reported for a species, divided by the total number of use categories reported for the most versatile species; and NT is the number of use subcategories reported for a given species divided by the number of use subcategories found in the most versatile species. We performed a non-parametric signed-ranks test (Wilcoxon test) to evaluate if there were significant differences between the literature and fieldwork matrices for (i) number of useful palm species, (ii) number of palm uses and (iii) average number of palm uses per species. All analyses were performed in JMP 10 (SAS institute).

### Ethics Statement

Approval for this study was granted by the Committee for Ethical Research of the Universidad Autónoma de Madrid (#48–922; PI Manuel J. Macía). We conducted our research outside of our countries of residence in association with the following local institutions: Instituto de Ciencias Naturales - Universidad Nacional de Colombia (Colombia); Pontificia Universidad Cátolica del Ecuador (Ecuador); Universidad Nacional Mayor de San Marcos (Peru); and Universidad Mayor de San Andrés (Bolivia). Before initiating data collection, we obtained oral informed consent in each case on a village level and then individually prior to each interview. This was done out of respect for the fact that some interviewees may lack reading or writing skills. The ethics committee of the Universidad Autónoma de Madrid approved this procedure. If the participant consented to being interviewed, consent was acknowledged by writing the date and name of the informant on the interview questionnaire. Informants could stop answering questions at any time, and they were aware that all information provided would be anonymized.

Palm collecting permits were obtained through the following authorities: Instituto Amazónico de Investigaciones Científicas Sinchi (Colombia); the Ministry of Environment (Ecuador); the Instituto Nacional de Recursos Naturales (Peru); and the Dirección General de Biodiversidad y Areas Protegidas (Bolivia). Field studies did not involve endangered or protected species.

## Supporting Information

Table S1 **Palm species arranged according to relative importance value indices (RI) based on fieldwork (F) and literature (L).** #-Uses, the total number of uses registered for each species during fieldwork (F) and in the literature (L); #-Informants, the total number of informants (out of 2201) who mentioned the species during interviews; and #-Publications, the number of ethnobotanical papers that mention the species out of the 255 publications reviewed.
(DOCX)
