# Supplementary material for: Ethnobotanical Knowledge Is Vastly Under-Documented in Northwestern South America
Source: PLoS One. 2014 Jan 9;9(1):e85794. doi: 10.1371/journal.pone.0085794 (PMC3887111; doi:10.1371/journal.pone.0085794)
Supplement: Table S1 — Palm species arranged according to relative importance value indices (RI) based on fieldwork (F) and literature (L). #-Uses, the total number of uses registered for each species during fieldwork (F) and in the literature (L); #-Informants, the total number of informants (out of 2201) who mentioned the species during interviews; and #-Publications, the number of ethnobotanical papers that mention the species out of the 255 publications reviewed. (DOCX) [file pone.0085794.s001.docx]

| **Species** | **RI** | | **#-Uses** | | **#-Informants** | **#-Publications** |
| --- | --- | --- | --- | --- | --- | --- |
|  | **F** | **L** | **F** | **L** | **F** | **L** |
| **Amazon ecoregion** |  |  |  |  |  |  |
| **Bactris gasipaes* var. *gasipaes* | 2.00 | 2.00 | 70 | 76 | 1138 | 109 |
| **Euterpe precatoria* | 1.98 | 2.00 | 62 | 89 | 1140 | 91 |
| **Oenocarpus bataua* | 1.93 | 2.00 | 75 | 107 | 1270 | 117 |
| **Mauritia flexuosa* | 1.90 | 1.90 | 60 | 95 | 1203 | 101 |
| **Astrocaryum chambira* | 1.75 | 1.60 | 54 | 60 | 696 | 68 |
| **Iriartea deltoidea* | 1.75 | 1.70 | 52 | 70 | 1152 | 79 |
| **Socratea exorrhiza* | 1.70 | 1.70 | 49 | 63 | 1182 | 69 |
| **Attalea phalerata* | 1.64 | 1.90 | 50 | 78 | 877 | 28 |
| **Oenocarpus mapora* | 1.63 | 1.70 | 38 | 50 | 1066 | 51 |
| **Attalea butyracea* | 1.61 | 1.60 | 46 | 37 | 872 | 26 |
| **Attalea maripa* | 1.59 | 1.70 | 67 | 61 | 756 | 31 |
| *Astrocaryum jauari* | 1.50 | 1.30 | 26 | 27 | 473 | 17 |
| **Astrocaryum aculeatum* | 1.46 | 1.50 | 32 | 39 | 299 | 17 |
| *Cocos nucifera* | 1.39 | 1.40 | 24 | 36 | 683 | 21 |
| *Mauritiella armata* | 1.31 | 0.90 | 21 | 14 | 421 | 12 |
|  |  |  |  |  |  |  |
| **Andes ecoregion** |  |  |  |  |  |  |
| **Attalea phalerata* | 2.00 | 1.30 | 40 | 21 | 268 | 1 |
| **Bactris gasipaes* var. *gasipaes* | 1.87 | 2.00 | 30 | 34 | 587 | 8 |
| **Socratea exorrhiza* | 1.70 | 1.10 | 26 | 10 | 239 | 3 |
| *Euterpe precatoria* | 1.54 | 0.60 | 24 | 6 | 203 | 1 |
| **Oenocarpus bataua* | 1.54 | 1.60 | 24 | 22 | 272 | 6 |
| *Ceroxylon vogelianum* | 1.44 | 0.60 | 25 | 6 | 208 | 5 |
| *Cocos nucifera* | 1.44 | 0.30 | 18 | 5 | 328 | 2 |
| *Prestoea acuminata* | 1.41 | 0.30 | 22 | 5 | 334 | 10 |
| *Mauritia flexuosa* | 1.31 | 0.60 | 16 | 6 | 110 | 4 |
| **Phytelephas aequatorialis* | 1.31 | 0.90 | 14 | 8 | 161 | 2 |
| *Socratea salazarii* | 1.31 | 0.00 | 17 | 0 | 63 | 0 |
| **Iriartea deltoidea* | 1.13 | 1.40 | 23 | 18 | 393 | 4 |
| *Aiphanes horrida* | 1.08 | 0.50 | 14 | 5 | 221 | 3 |
| *Astrocaryum chambira* | 1.08 | 0.20 | 15 | 2 | 59 | 1 |
| *Astrocaryum murumuru* | 1.08 | 0.00 | 12 | 0 | 62 | 0 |
|  |  |  |  |  |  |  |
| **Chocó ecoregion** |  |  |  |  |  |  |
| **Bactris gasipaes* var. *gasipaes* | 2.00 | 1.60 | 14 | 24 | 309 | 13 |
| **Astrocaryum standleyanum* | 1.82 | 1.40 | 27 | 27 | 194 | 18 |
| **Iriartea deltoidea* | 1.75 | 1.40 | 37 | 17 | 292 | 14 |
| **Wettinia quinaria* | 1.57 | 1.60 | 22 | 20 | 282 | 13 |
| **Oenocarpus bataua* | 1.57 | 1.20 | 28 | 18 | 243 | 15 |
| **Cocos nucifera* | 1.56 | 2.00 | 24 | 30 | 313 | 11 |
| **Oenocarpus mapora* | 1.50 | 1.10 | 22 | 14 | 171 | 8 |
| **Attalea colenda* | 1.42 | 1.00 | 16 | 12 | 84 | 8 |
| *Elaeis guineensis* | 1.35 | 0.20 | 14 | 1 | 220 | 1 |
| *Attalea allenii* | 1.28 | 0.70 | 18 | 7 | 173 | 6 |
| **Phytelephas aequatorialis* | 1.28 | 1.20 | 13 | 14 | 52 | 5 |
| *Welfia regia* | 1.24 | 0.60 | 17 | 8 | 181 | 6 |
| **Manicaria saccifera* | 1.23 | 0.90 | 21 | 10 | 167 | 9 |
| *Bactris gasipaes* var. *chichagui* | 1.21 | 0.00 | 14 | 0 | 42 | 0 |
| *Asterogyne martiana* | 1.13 | 0.00 | 13 | 0 | 134 | 0 |

* Shared species between fieldwork and bibliographic results with high relative importance
